# Role of latent female genital tuberculosis in recurrent early pregnancy loss: A retrospective analysis

**DOI:** 10.18502/ijrm.v17i12.5799

**Published:** 2019-12-30

**Authors:** Bishista Bagchi, Siddhartha Chatterjee, Rajib Gon Chowdhury

**Affiliations:** Department of Reproductive Medicine, Calcutta Fertility Mission, Kolkata, India.

**Keywords:** Female genital tuberculosis, Recurrent pregnancy loss, Endometrium, Implantation.

## Abstract

**Background:**

Latent Female Genital tuberculosis (FGTB) or tubercular infestation is prevalent in Southeast Asia and even the presence of tubercular bacilli in the genital tract is becoming an important factor for reproductive failure. An immature endometrium becomes non-receptive, preventing implantation or rejection of implanted embryo in early months, resulting in recurrent pregnancy loss (RPL) in association with other factors.

**Objective:**

To detect the underlying causes of RPL in addition to the proven causes like uterine cavity defects, thrombophilia, chromosomal abnormalities, etc.

**Materials and Methods:**

317 women with RPL, enrolled over a period of 60 months (January 2014 to December 2018) conducted at Calcutta Fertility Mission in the present study. They were grouped in A, B, and C and undergone routine tests for the same along with the PCR test with an endometrial aspirate.

**Results:**

Patients with only latent FGTB (Group A), patients with FGTB and associated factors (Group B), and patients with other causes of RPL (other than latent FGTB) (Group C) were34.4%, 42.3%, and 23.3% respectively. About 29.36%, 47.01%, and 21.62%of the patients had achieved pregnancy in Group A, B, and C, respectively. The rate of miscarriage was high in both Groups A and B, affected with latent FGTB, and live-birth was higher (75%) in Group C that did not have tubercular involvement of the genital tract.

**Conclusion:**

The tubercular infestation or latent FGTB as per our study appears to be a very important cause of RPL in patients with recurrent “unexplained” miscarriage. It should be treated adequately at an early stage to prevent permanent damage to pelvic organs and restore reproductive health in women.

## 1. Introduction

Female genital tuberculosis (GTB) is typically detected in 18-19% of Indian women of the reproductive age groups as per a study conducted in 2008, which increased to 19-30% in 2015, affecting the pelvic organs by different routes, often during investigating for infertility (1). It is difficult to find out the actual incidence of GTB as many cases are asymptomatic and diagnosed incidentally. Although the frank Extra-pulmonary tuberculosis (EPTB) in the form of GTB is prevalent in Southeast Asia, the mere presence of tubercular bacilli in the genital tract which may otherwise be called as latent female genital tuberculosis (FGTB)infestation is becoming more and more important as a causative factor for reproductive failure (2, 3). Implantation is one of the important early stages for the initiation of successful pregnancy that involves formation of matured embryo and receptive endometrium. An immature endometrium becomes non-receptive that does not allow implantation and rejects the implanted early embryo from its nidation site. Endometrial development may be affected by tubercular infestation that invites harmful cytokines in the decidua. It has been observed that sub-endometrial vascularity decreases due to the involvement of basal layer of the endometrium in latent FGTB as evident by pioneer work (4). The possibility of vascular micro thrombus formation following the tubercular assault of endometrium leads to implantation failure and miscarriage. Tuberculous endometritis is found in 80% of patients, whereas tuberculous salpingitis and oophoritis are seen in around 8% of them (5, 6). However, according to few, the endometrium appears to be the most commonly involved site in EPTB (7). The incidence of pregnancy and live birth following is seen to be quite low and the possibility of ectopic pregnancy increases or results in loss of pregnancy (8, 9).

The purpose of this study was to detect the underlying causes of recurrent pregnancy loss (RPL) in addition to the causes like uterine cavity defects, thrombophilia, chromosomal abnormalities, etc., so far mentioned in literature.

## 2. Materials and Methods

A total of 317 woman aged range between 20-45 yr old who were referred to Department of Reproductive Medicine at Calcutta Fertility Mission, over a period of 60 months (January 2014 to December 2018) were enrolled in this observational study. The inclusion criteria were woman who had two or more recurrent pregnancy losses, women who had diagnosed uterine malformations, chromosomal defects, thrombophilia etc. were excluded.

Women were divided into three groups: Groups A, B, and C in which Group A (only TB-PCR Positive, n = 109) included patients with RPL who had only latent FGT Bas the etiological factor of the losses among the factors screened in this study. Group B (TB-PCR Positive, n = 134) consisted of women with recurrent miscarriage who were found to have tubercular bacilli along with any of the other factors as etiology. Group C (TB-PCR-Negative, n = 74) included patients who did not have any tubercular infestation but some other etiology for RPL. They had undergone routine tests for the same along with the PCR test with an endometrial aspirate.

### Ethical consideration

The Ethical Committee of Calcutta Fertility Mission has given clearance for the retrospective study of a prospective database on 02/01/2019 (code: CFM/2019/024). Written informed consent has been obtained from all women who participated in the study.

### Statistical analysis

The obtained results were analyzed using SPSS Statistics software (Statistical Package for the Social Sciences, version 17.0, SPSS Inc, Chicago, Illinois, USA). The comparison between groups was evaluated using the Pearson's Chi-Square test and Fischer's exact test. A P value of < 0.05 was considered to be statistically significant.

## 3. Results

A total of 317 patients included in this study were divided into three groups as follows: Group A consisted of 109 patients (34.4%), Group B included 134 patients (42.3%), and Group C had 74 patients (23.3%). Both the age of the patient and their obstetric score did not have any statistical significance in the present study. 76.6% had latent FGTB detected by PCR test (TB-PCR positive). On a descriptive statistical analysis, it was observed that the latent FGTB was an important cause of RPL irrespective of association with the other etiological factors, age, or the obstetric score (Tables I, II). Other causes of RPL like overt diabetes, hypothyroidism or hyperthyroidism, hyperandrogenism, hyperprolactinemia, low ovarian reserve, hyperhomocysteinemia, antiphospholipid antibody syndrome, and toxoplasmosis were analyzed (Table III).

Both impaired Glucose Tolerance Test and abnormal levels of TSH were not seen to be a statistically significant cause of RPL (p = 0.618). Though we found that the patients suffering from RPL have increased testosterone or decreased AMH levels with or without associated latent FGTB, these parameters were not statistically significant (p = 0.377). Even increased levels of Serum Homocysteine was not found to have any statistical (p = 0.496).

Group C participants who had hyperprolactinemia as the only etiology of RPL were more than the participants of Group B who had increased levels of prolactin associated with latent FGTB. In both groups, B and C, toxoplasmosis was seen to be a statistically significant factor for causing RPL. Anti-cardiolipin antibodies and Lupus anticoagulant time also did not have any statistical significance as the RPL etiology. Among these three groups the clinical pregnancy rate (CPR), live birth rate (LBR), and miscarriage rate have been calculated and compared. CPR within next 12 months, after taking anti-tubercular drugs for six months, was more in Group B when compared to Groups A and C and was statistically significant. Also, LBR and miscarriage rate was statistically significant (Table IV). The rate of miscarriage was high in both Groups A and B compared to Group C.

**Table 1:** Age of women in groups


**Age**	**Group**			**Total**	**P-value**
	**Group A**	**Group B**	**Group C**		0.060
20-25	27 (24.77)	16 (11.94)	17 (22.97)	60 (18.93)	
26-30	40 (36.7)	59 (44.03)	19 (25.68)	118 (37.22)	
31-35	29 (26.61)	37 (27.61)	25 (33.78)	91 (28.71)	
36-40	9 (8.26)	19 (14.18)	12 (16.22)	40 (12.62)	
41-45	4 (3.67)	3 (2.24)	1 (1.35)	8 (2.52)	

Data presented as n (%); Pearson's Chi-Square test					

**Table 2:** Obstetric score of participants


**Obstetric score**	**Group**			**Total**	**P-value**
	**Group A**	**Group B**	**Group C**		
P (0+2)	23 (21.1)	43 (32.09)	29 (39.19)	95 (29.97)	0.064
P (0+3)	60 (55.05)	59 (44.03)	27 (36.49)	146 (46.06)	
P (0+4)	11 (10.09)	13 (9.7)	5 (6.76)	29 (9.15)	
P (0+5)	2 (1.83)	9 (6.72)	1 (1.35)	12 (3.79)	
P (0+6)	4 (3.67)	1 (0.75)	1 (1.35)	6 (1.89)	
P (0+7)	1 (0.92)	1 (0.75)	3 (4.05)	5 (1.58)	
P (1+2)	3 (2.75)	3 (2.24)	3 (4.05)	9 (2.84)	
P (1+3)	2 (1.83)	4 (2.99)	2 (2.7)	8 (2.52)	
P (1+4)	3 (2.75)	0 (0)	1 (1.35)	4 (1.26)	
P (2+2)	0 (0)	1 (0.75)	2 (2.7)	3 (0.95)	

**Table 3:** Participants etiology


	**Group A**	**Group B**	**Group C**	**P-value**
Pcr/Mtb	109 (100)	134 (100)	0 (0)	< 0.001
Gtt	0 (0)	42 (31.34)	14 (18.92)	0.053
Tsh	0 (0)	20 (14.93)	13 (17.57)	0.618
Toxo	0 (0)	4 (2.99)	12 (16.22)	0.001
Aca	0 (0)	9 (6.72)	3 (4.05)	0.545
La	0 (0)	5 (3.73)	5 (6.76)	0.339
St	0 (0)	32 (23.88)	16 (21.62)	0.711
Amh	0 (0)	34 (25.37)	23 (31.08)	0.377
Prl	0 (0)	15 (11.19)	20 (27.03)	0.003
Homocysteine	0 (0)	7 (5.22)	2 (2.7)	0.496

Data presented as n (%); Fischer's exact test				
PCR/MTB: Polymerase chain reaction/ mycobacterium tuberculosis; GTT: Glucose tolerance test; TSH: Thyroid stimulating hormone; TOXO: Toxoplasmosis; ACA: Anti-cardiolipin antibody; LA: Lupus anticoagulant; ST: Serum testosterone; AMH: Anti-Mullerian hormone; PRL: Prolactin				

**Table 4:** Clinical pregnancy rate, live birth rate, and miscarriage rate in groups


	**Group A**	**Group B**	**Group C**	**Total**	**P-value**
Clinical pregnancy	32 (29.36)	63 (47.01)	16 (21.62)	111 (35.02)	< 0.001
Live birth	12 (37.5)	22 (34.92)	12 (75)	46 (41.44)	0.013
Miscarriage	20 (62.5)	39 (61.9)	3 (18.75)	62 (55.86)	0.005

Data presented as n (%); Pearson's Chi-Square test					

## 4. Discussion

Repeated miscarriage appears to be a curse in a woman's reproductive career. It becomes difficult to form a work-up to detect the cause of RPL in the early months as a continuation of pregnancy involves many factors. The factors may be genetic factors, anatomical causes (uterine cavity defect), hormonal causes, or miscellaneous like infections and immunological factors. In this study, uterine defects and ectopic pregnancy have been excluded, and a focus is placed on other factors. Routine tests which involve factors mentioned previously have been performed in all patients and one new test included in the miscellaneous group has been performed to detect a tubercular infestation of the endometrium as a causative factor of RPL (10). It has been observed that quite a large number of cases of RPL are affected with latent FGTB or a tubercular infestation of the endometrium as a sole or strongly associated factor for the development of such an unfortunate situation.

The prevalence of FGTB in developing countries is high among infertile patients with tubal factor involvement, involving almost 90%; with 82.9% having endometrial involvement too (11).

It has been seen that 92.3% of infertile women without tubal defects who had been treated with anti-tubercular drugs (ATD) based on a positive endometrial aspirate TB-PCR test had spontaneous conception within 12 months of treatment (12). Similarly, in our study, patients with RPL with secondary infertility who otherwise had no other demonstrable cause of pregnancy loss other than latent FGTB, 29.36% of patients had conceived spontaneously during or within 12 months of ATD administration. 47.01% of the patients in our study had spontaneous conception after a treatment with ATD and any other associated etiological factor for RPL. Anti-tubercular treatment (ATT) should be given in early disease if the patient had raised ESR ( ≥ 20 mm/first hr), Mantoux test positive (induration ≥ 10 mm), hysterosalpingography picture or ultrasonological picture suggestive of GTB. Hence the chances of pregnancy can be enhanced and irreversible damage to the endometrium and especially fallopian tubes can be prevented and there will be increased chances of conception (13).

In our study, 16.22% of patients had toxoplasmosis as the sole factor for RPL, whereas 2.99 % of them had toxoplasmosis associated with latent FGTB, which complies with other studies (14, 15). Hyperprolactinemia in initial stage of follicular growth may hinder secretion of progesterone which eventually result in luteal phase defects and RPL, and the treatment may reduce the rate of miscarriage in a subsequent pregnancy in these women which is similar to our findings (16).

In recent studies, only 36.7% patients with GTB have been noted to achieve pregnancy after ATD treatment which concludes that the outcome of infertility in GTB is not very optimistic and IVF-ET need to be considered in such cases (8). In this study, CPR and LBR were relatively better being 29.36% and 37.5%, respectively, in Group A and 47.01% and 34.92%, respectively, in Group B.

In our study, the rate of miscarriage in an ongoing pregnancy was high in both Group A (62.5%) and Group B (61.9%); compared to 18.75% in Group C that correlates with other studies (17) which conclude that in women with GTB, the CPR per cycle was low and spontaneous abortion was high. Women with GTB were seen to represent a less favorable group within other tubal factor patients when treated with IVF-ET.

Low-grade inflammation might lead to minimal structural and functional alteration of the pelvic organs as observed in Mycobacterium tuberculosis infestation. Mycobacterium colonization in the endometrium, what has been mentioned as the tubercular infestation, may be a cause of reproductive failure too (3). An important cause of implantation failure is the immunological factor and improving endometrial receptivity in the course of fertility management by improving success rates. The evaluation of implantation markers is necessary to detect occult endometrial assault. The most offensive cytokine for the loss of pregnancy is TNF-α. TNF-α, and anti-cardiolipin antibody levels are seen to be potential diagnostic markers and they exhibit significant role in prognosis of RPL patients (18). An increased prevalence of IFN-γ has been shown in endometrial aspirate in TBPCR positive cases. Hence, IFNγ showed a possibility to become an important clinical indicator of endometrial hostility followed by IL2 and on treatment by ATD improved reproductive outcome indicating the ill effect is reversible, as in contrast to tubercular infection (3). Key endometrial genes expression along with gene or stem cell-based therapies may be incorporated to improve implantation (19). Tubercular infestation or LGTB as per our study appears to be a very important cause in patients with apparently unexplained RPL following routine tests. It appears that the exclusion of tubercular infestation should be one of the most relevant investigations in cases of RPL.

##  Conflict of Interest

There is no conflict of interest in between the authors involved in this study.

## References

[1] Sharma JB., Sharma E., Sharma S., Dharmendra S. (2018). Female genital tuberculosis. *Revisited. Indian J Med Res*.

[2] Mahajan N., Naidu P., Kaur S. (2016). Insight into the diagnosis and management of subclinical genital tuberculosis in women with infertility. *Journal of Human Reproductive Sciences*.

[3] Datta A., Chaudhuri A., Chatterjee S., Chowdhury R., Bhattacharya B. (2018). Role of endometrial cytokines of the female genital tract tuberculosis in the context of infertility. *BLDE University Journal of Health Sciences*.

[4] Ravi B., Gupta D. O., Pooja S., Bokariya P. (2010). Alveolar rhabdomyosarcoma of oral cavity##hssm###8212;a rare case. *Al Ameen Journal of Medical Sciences*.

[5] Shahzad S. (2012). Investigation of the prevalence of female genital tract tuberculosis and its relation to female infertility: An observational analytical study. *Iranian Journal of Reproductive Medicine*.

[6] Singh S., Gupta V., Modi S., Rana P., Duhan A., Sen R. (2010). Tuberculosis of uterine cervix: a report of two cases with variable clinical presentation. *Tropical Doctor*.

[7] Ishrat S., Fatima P. (2015). Genital tuberculosis in the infertile women - an update. *Mymensingh medical journal : MMJ*.

[8] Yue J., Zhang B., Wang M., Yao J., Zhou Y., Ma D., Jin L. (2019). Effect of antitubercular treatment on the pregnancy outcomes and prognoses of patients with genital tuberculosis. *Frontiers of Medicine*.

[9] Mondal S. K., Dutta T. K. (2009). A ten year clinicopathological study of female genital tuberculosis and impact on fertility. *Journal of Nepal Medical Association*.

[10] Paine S. K., Basu A., Choudhury R. G., Bhattacharya B., Chatterjee S., Bhattacharya C. (2018). Multiplex PCR from Menstrual Blood: A Non-Invasive Cost-Effective Approach to Reduce Diagnostic Dilemma for Genital Tuberculosis. *Molecular Diagnosis & Therapy*.

[11] Al eryani A. A., Abdelrub A. S., Al Harazi A. H. (2019). Genital tuberculosis is common among females with tubal factor infertility: Observational study. *Alexandria Journal of Medicine*.

[12] Jindal U. N., Verma S., Bala Y. (2012). Favorable infertility outcomes following anti-tubercular treatment prescribed on the sole basis of a positive polymerase chain reaction test for endometrial tuberculosis. *Human Reproduction*.

[13] Naredi N., Talwar P., Narayan N., Rai S., Vardhan Sh., Panda S. (2014). Spontaneous conception following anti-tubercular treatment for sub-fertile women with multiple imaging markers suggesting genital tuberculosis. *Fertility Science and Research*.

[14] Vado-Solís I. A., Suárez-Solís V., Jiménez-Delgadillo B., Zavala-Velázquez J. E., Segura-Correa J. C. (2013). *Toxoplasma gondii* presence in women with spontaneous abortion in Yucatan, Mexico. *The Journal of Parasitology *.

[15] Nasirpour H., Azari Key Y., Kazemipur N., Majidpour M., Mahdavi S., Hajazimian S., Issazadeh A., Taefehshokr S. (2017). Association of Rubella, Cytomegalovirus, and Toxoplasma Infections with Recurrent Miscarriages in Bonab-Iran: A Case-Control Study. *Gene, Cell and Tissue*.

[16] El Hachem H., Crepaux V., May-Panloup P., Descamps P., Legendre G., Bouet P. (2017). Recurrent pregnancy loss: current perspectives. *International Journal of Women's Health*.

[17] Angeline Grace G., Bella Devaleenal D., Natrajan M. (2017). Genital tuberculosis in females. *Indian Journal of Medical Research*.

[18] Van Niekerk E. C., Siebert I., Kruger T. F. (2013). An evidence-based approach to recurrent pregnancy loss. *South African Journal of Obstetrics and Gynaecology*.

[19] Cakmak H., Taylor H. S. (2011). Implantation failure: molecular mechanisms and clinical treatment. *Human Reproduction Update*.

